# Insects in temperate urban parks face stronger selection pressure from the cold than the heat

**DOI:** 10.1002/ece3.11335

**Published:** 2024-08-19

**Authors:** Jelena Bujan, Cleo Bertelsmeier, Ana Ješovnik

**Affiliations:** ^1^ Division for Marine and Environmental Research Ruđer Bošković Institute Zagreb Croatia; ^2^ University of Lausanne Lausanne Switzerland; ^3^ Croatian Myrmecological Society Zagreb Croatia; ^4^ Department of Entomology, National Museum of Natural History Smithsonian Institution Washington District of Columbia USA; ^5^ Institute for Environment and Nature Zagreb Croatia

**Keywords:** ants, CTmax, CTmin, ecophysiology, insects, microclimates, peri‐urban, thermal tolerance, urban green spaces

## Abstract

Urban areas experience higher temperatures compared to rural areas and as such, are increasingly considered places of acclimatization and adaptation to warming. Small ectotherms, such as insects, whose body temperature rises with habitat temperature, are directly affected by temperature changes. Thus, warming could have a profound effect on insect behavior and physiology. To test if the urban heat island effect drives higher thermal tolerance and activity changes, we used globally distributed and abundant insects—ants. We measured the heat and cold tolerance of 14 ant species distributed across urban and peri‐urban areas. As thermal traits are often correlated with ant foraging, we measured foraging activity during three consecutive years across eight sites. Contrary to our prediction, ants exposed to the urban heat island effect did not have a higher heat tolerance than peri‐urban ants. Instead, cold tolerance varied across habitats, with ants from the cooler, peri‐urban habitats being able to tolerate lower temperatures. We recorded the same pattern of invariant heat and higher cold tolerance for ants in the canopy, compared to ground nesting ants. Ant activity was almost 10 times higher in urban sites and best predicted by cold, not heat tolerance. These unexpected results suggest that we need to rethink predictions about urban heat islands increasing insect heat tolerance in urban habitats, as cold tolerance might be a more plastic or adaptable trait, particularly in the temperate zone.

## Graphical abstract

We tested the impact of the urban heat island effect on the thermal tolerance and activity of ants. We found no difference in ant heat tolerance across urban and peri‐urban sites, despite urban sites being warmer. Instead, cold tolerance followed habitat temperatures and was higher in cooler, peri‐urban sites. Taken together, our results challenge assumptions about the effect of urban heat islands on insect heat tolerance.

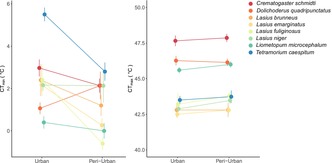

## 1 INTRODUCTION

Urbanization is increasing globally (Seto et al., 2012). Cities are rich with impervious surfaces, such as asphalt and concrete, resulting in higher temperatures than less urbanized habitats (Deilami et al., 2018). This urban heat island effect could be particularly detrimental for small ectotherms, such as insects, which are the most diversified taxonomic group on Earth. In disturbed and hot urban habitats, urban green areas are becoming increasingly important for biodiversity protection (Fattorini & Galassi, 2016; Hall et al., 2017) and temperature cooling (Aram et al., 2019).

Urban green spaces across the city's thermal gradient allow for field testing of species adaptations to high temperatures as they offer an overlapping set of species—many plant and animal species are present across the entire gradient of urban green spaces—enabling direct intraspecific comparisons (Pinho et al., 2016). But there is also a vertical thermal gradient in urban green spaces containing trees: the canopy and the ground. The tropical canopy is on average warmer and drier than the understory, and it contains insects adapted to such conditions with higher heat tolerance and desiccation resistance (Bujan et al., 2016; Kaspari et al., 2015). However, the canopy in the temperate zone is hotter than the ground during the hottest parts of the day and cooler during the coldest (Morecroft et al., 1998), and we do not know if thermal tolerances of insects follow this trend. If this is the case, then canopy insects are expected to have higher heat and cold tolerances.

Rates of phenotypic change in animals and plants globally seem to be greater in urban than natural or rural areas (Alberti et al., 2017). Because of this, urban habitats are considered thermal adaptation hubs and laboratories of evolution (Szulkin et al., 2020). Some studies found that urban insects exhibit higher heat tolerances than their rural conspecifics (Angilletta et al., 2007; Diamond et al., 2017), which can be associated with the evolution of higher thermal tolerance in urban areas (Martin et al., 2019). Urbanization can also lead to an increase in body size of moths and bumblebees (Merckx et al., 2018; Theodorou et al., 2021) and an increased plasticity of heat tolerance in ants (Diamond & Martin, 2021).

To test insects' response to increased temperatures, we studied thermal tolerances, abundance, and activity of ants. Since ant activity is directly governed by temperature and limited by their thermal tolerance (Cerdá et al., 1998; Roeder et al., 2018), thermal adaptations can have direct effects on foraging success and colony fitness. Ant colonies are usually semi‐sessile, perennial, with restricted foraging ranges and limited gene flow. This makes them an ideal taxon to monitor long‐term environmental change and well suited to studies of microclimate adaptations in comparison to solitary, more mobile insects.

Studies combining ant physiology and behavior across thermal gradients and timeframes—from yearly to seasonal variations (Bujan et al., 2022; Diamond et al., 2012; Roeder et al., 2018) help us understand community level responses to temperature, yet they usually focus only on heat tolerance (Youngsteadt et al., 2023). Additionally, studies of ant heat and cold tolerance in urban and rural habitats usually focus on one (Angilletta et al., 2007; Diamond et al., 2017; Martin et al., 2019) or two species (Warren et al., 2018). Our study is the first to examine thermal tolerance of ant communities across horizontal and vertical urban thermal gradients. We relate these physiological changes to yearly community‐level changes in ant abundance, richness, and activity.

We tested the thermal adaptation hypothesis, which posits that thermal tolerance corresponds to environmental temperatures (Roeder et al., 2021). We predicted that warmer, urban, sites will harbor ants with higher heat and lower cold tolerance at the community level and at the population level. We compared canopy and ground nesting ants from distinct park types: urban, city center parks that are smaller, warmer, and more isolated, and peri‐urban sites which are cooler and less isolated, located at the edge of the city. Based on the predictions of the thermal adaptation hypothesis, and knowledge of temperature fluctuations in the temperate zone canopy (Morecroft et al., 1998), we predicted that canopy ants will have higher heat and cold tolerance. We then test whether temporal changes in ant abundance and richness exist across eight urban and peri‐urban sites. We predict that temperature will be the driver of temporal changes in abundance and richness, as lower temperatures are expected to reduce ant activity resulting in both lower abundance and richness.

Lastly, we tested whether ant activity in urban and peri‐urban areas can be predicted by species' thermal tolerances, as is generally the case for natural habitats (Cerdá et al., 1998). Ant foraging is fine tuned to environmental temperatures and closely tied to ants' upper thermal limits even in cities (Youngsteadt et al., 2023). In some disturbed habitats, both abundance and activity can be best explained by the interaction of heat tolerance and environmental temperature (Boyle et al., 2021). In urban habitats, which are more disturbed than rural habitats, foraging activity can also be driven by the most dominant ant species (Youngsteadt et al., 2015). But since the activity of such dominant ants is also governed by their heat tolerance (Roeder et al., 2018), we did not expect that species dominance would interfere with our physiological predictions. Here, we combine long‐term data of changes in abundance and activity with upper and lower thermal limits across different microhabitats to understand thermal adaptations caused by urban heat islands.

## 2 MATERIALS AND METHODS

We sampled ants in the city of Zagreb, Croatia (45.80° N, 15.99° E, elevation 145 m) for 3 years. The city of Zagreb has a population size of about 800,000 (Šiško & Polančec, 2019). The climate in Zagreb is a temperate continental climate with warm summers (Zaninovic et al., 2008). The average temperature in the warmest month of July is 22.1°C, and in the coldest month of February is 3.4°C (1949–2018) (Bonacci et al., 2021). The number of summer days with temperatures above 25°C has been increasing in the urban area of Zagreb for 8 days every 10 years (Nimac et al., 2021). Temperature fluctuations in mean annual temperatures are less pronounced, with an average yearly temperature of 12.1°C (Bonacci et al., 2021). We collected ants at eight locations in Zagreb urban parks. Five sites were in the city center, we refer to those as urban sites henceforward. Three sites were located in a large protected forested area (about 4 km^2^) in a less urbanized part of the city, and we refer to those as peri‐urban sites (Figure S1).

### 2.1 Climatic measurements

We measured microclimates at two locations in the center and two in the peri‐urban area (Figure S1). At each site, we placed one data logger (EL‐USB‐2 LascarEasy Log) on the ground in the shade of a hedge bush. Loggers recorded temperature and relative humidity (RH) at 5‐min intervals during the sampling of ants for critical thermal limits measurements (21st June–25th July 2021).

Additionally, we used daily air temperature averages for June of each sampling year (2019–2021) recorded by the Croatian Hydrological and Meteorological Service (DHMZ) to test temperature effect on ant abundance and richness in pitfalls across 3 years of sampling. These data were recorded ~2 m above ground at two weather stations: urban measuring station Grič (45.81468, 15.97196), 600–1000 m away from the surveyed city parks, and peri‐urban station Maksimir (45.8213, 16.02594), 500–1100 m away from the surveyed peri‐urban sites.

### 2.2 Measuring critical thermal limits

Critical thermal limits (CTLs) are temperatures at which animals lose voluntary muscle control (Lutterschmidt & Hutchison, 1997). Although the daily temperatures ants experience in their habitats are rarely close to their critical thermal maximum (CT_max_), it is still a useful proxy for temperature preference. Ant species with higher CT_max_ are going to forage during hotter parts of the day (Cerdá et al., 1998; Roeder et al., 2018). CT_max_ has been used in ant research at local and global scales (Roeder et al., 2021), as well as for predicting ectotherm responses to climate change (Sunday et al., 2014). To measure CTLs, we collected ants at the same sites used to measure microclimates during late June and early July of 2021 (Figure S1). We collected foragers outside of their nests with aspirators between 8:00 and 13:00 and tested them immediately after collection to avoid potential acclimation (a maximum 4 h elapsed before testing). We chose this sampling window to make sure we captured all commonly foraging ant species, because even though there is not a strong preference for foraging time, species will start foraging only when it gets warm enough, which can be later in the day (e.g., *Colobopsis truncata*). We made sure to collect ants from distinct colonies, by collecting foragers in the vicinity of different nests. To measure ant CTLs, we used a chilling/heating dry bath (Torrey Pines Scientific EchoTherm™ IC50; advertised accuracy ±0.2°C) and a standardized ramping protocol for recording ant thermal limits (Bujan et al., 2020). In short, we tested workers individually in 1.5 mL microcentrifuge tubes. Critical thermal maximum (CT_max_) measurements started at 36°C, and we increased the temperature by 2°C every 10 min. We inspected and rotated each tube before the next temperature increase, to determine at which temperature ants lost muscle control. To measure the critical thermal minimum (CT_min_), we started the trials at 16°C and used the same procedure and the same rate, but of temperature decrease.

We tested ten workers per colony, five workers for CT_min_ and five for CT_max_ measurement. Species were replicated with 1–7 colonies at urban and 1–6 at peri‐urban sites (median = 3 for both sites). Thus, if a species was present at both urban and peri‐urban sites, it was represented by six unique colonies (mean ± SE: 6.1 ± 1.1) and 60 workers (30 workers for each CTL measurement). In total, we measured critical thermal limits of 14 ant species in 10 different genera, 7 species were canopy and 7 were ground nesting ants. Ten species came from urban, and 13 from peri‐urban sites (Table S1).

### 2.3 Measuring ant activity

Ant activity in Zagreb urban parks is highest in June (Ješovnik & Bujan, 2021). Thus, we measured ant activity in June for three consecutive years (2019–2021) to compare the differences in ant activity across urban and peri‐urban sites. We used baits to measure both ant recruitment (number of ants at baits) and ant activity (occurrence at baits). As a bait, we used a piece of cotton soaked in honey water placed at the bottom of 2 mL Eppendorf tube. We distributed 15 baits per site along a linear transect with 2 m between baits. Using a portable IR thermometer (Fluke 62Max, Fluke Corporation, Everett, Washington, USA) we measured ground surface temperature at the beginning of each baiting trial and 1 h later, upon bait collecting. We transferred ants from each bait to a separate vial with 95% EtOH for later identification. During 3 years of sampling, we set up 360 baits, which attracted a total of 2543 individual ants from 8 species in 7 different genera (Table S1).

### 2.4 Measuring abundance and richness

To record differences in species abundance and richness across sites, we used pitfall traps in June for three consecutive years (2019–2021). At each of the eight locations, we set up ten traps in two linear transects of five traps. The distance between the traps was 3 m, and between the transects, it was a minimum of 10 m. Our traps were 50 mL Falcon tubes filled with ~15 mL of 60% ethanol and a drop of glycerol. Traps were left open for 48 h, after which we stored the samples in 95% ethanol. We used a total of 240 pitfall traps during 3 years of sampling, which collected a total of 1687 individual ants from 20 species and 13 genera (Table S1). None of the recorded species were non‐native which indicates that alien ant species are either absent in studied parks or they are currently at very low population densities (Ješovnik & Bujan, 2021). We identified all ants by species using the stereomicroscope (XTL3400D) and taxonomical keys (Radchenko & Elmes, 2010; Seifert, 2018; Wagner et al., 2017), and an online ant specimen database Ant Web (https://www.antweb.org). All specimens are stored in the Croatian Myrmecological Society ant collection in Zagreb.

### 2.5 Data Analysis

#### 2.5.1 Microclimatic differences

We tested if average daily temperatures depended on site (urban vs. peri‐urban) and time of day (day vs. night). To do so, we divided our soil surface measurements into daytime (5:30–20:30) and nighttime (20:35–5:25) based on sunrise and sunset times in Zagreb during June and July. The same type of model with site and time of day as predictors was fitted to examine differences in relative humidity. We used the *glmmTMB* function to fit the models (Brooks et al., 2017).

#### 2.5.2 Critical thermal limits

To test for differences in ant CTLs, we used separate generalized linear mixed effect models for CT_max_ and CT_min_. Across 14 tested species, we analyzed how CTLs change across different sites (urban vs. peri‐urban) and habitats (arboreal vs. ground) using colony averages of 5 workers. All models had two random factors: (1) species to control for species level differences, and (2) colony to control for intra‐specific variability. Each CTL model had two categorical fixed effects (site and habitat) and two random effects (species, colony).

#### 2.5.3 Activity analysis

To analyze differences in ant activity at baits, we used average ground temperature, sampling year, site, and ant thermal tolerance (CT_max_, CT_min_, and T_range_) as fixed factors. We calculated the range of temperatures that each species in each population can tolerate (T_range_) as the difference between CT_max_ and CT_min_. Bait identity was used as a random factor to control for repeated measurements. To analyze ant recruitment, we analyzed the total number of ants at baits using the ‘nbinom2’ family for zero‐inflated data. We built separate models for each thermal trait and used model comparison to test whether CT_max_, CT_min_ or T_range_ best predict ant occurrence and recruitment. The optimal model with the lowest AIC (>2AIC from other models) was the model with CT_min_ as a fixed factor, so we reported those results below. The high number of ants at baits can reflect ant recruiting success and colony vicinity, so to minimize this potential sampling artifact we also used occurrence of ants at baits (presence or absence). A separate model with presence‐absence data for occurrence used the same predictors as for recruitment but with a binomial family distribution.

#### 2.5.4 Measuring abundance and richness

We used log transformed abundance and richness data of ants recorded in pitfall traps to test if they are affected by the sampling year, site, or average June temperature. We used a generalized linear mixed model with a trap number as a random factor, as it accounted for the repeated measures at each of the sites in three consecutive years. We used separate models to analyze the changes in species richness and abundance. In the model examining species richness, we used the wooded area of the park as a predictor variable instead of site, as we previously found that the wooded area best predicts ant richness across Zagreb parks (Ješovnik & Bujan, 2021).

We checked for overdispersion of all models using diagnostic plots in the DHARMa package (Harting, 2021). We calculated model estimates and post‐hoc comparisons with the *emmeans* R package (Lenth et al., 2020). All data were analyzed with the R statistical program (R Core Team, 2023). The data supporting the findings of this study will be publicly available in Figshare upon publication: https://figshare.com/s/b466915a1d1ce115d441.

## 3 RESULTS

### 3.1 Microclimatic differences

**FIGURE 1:**
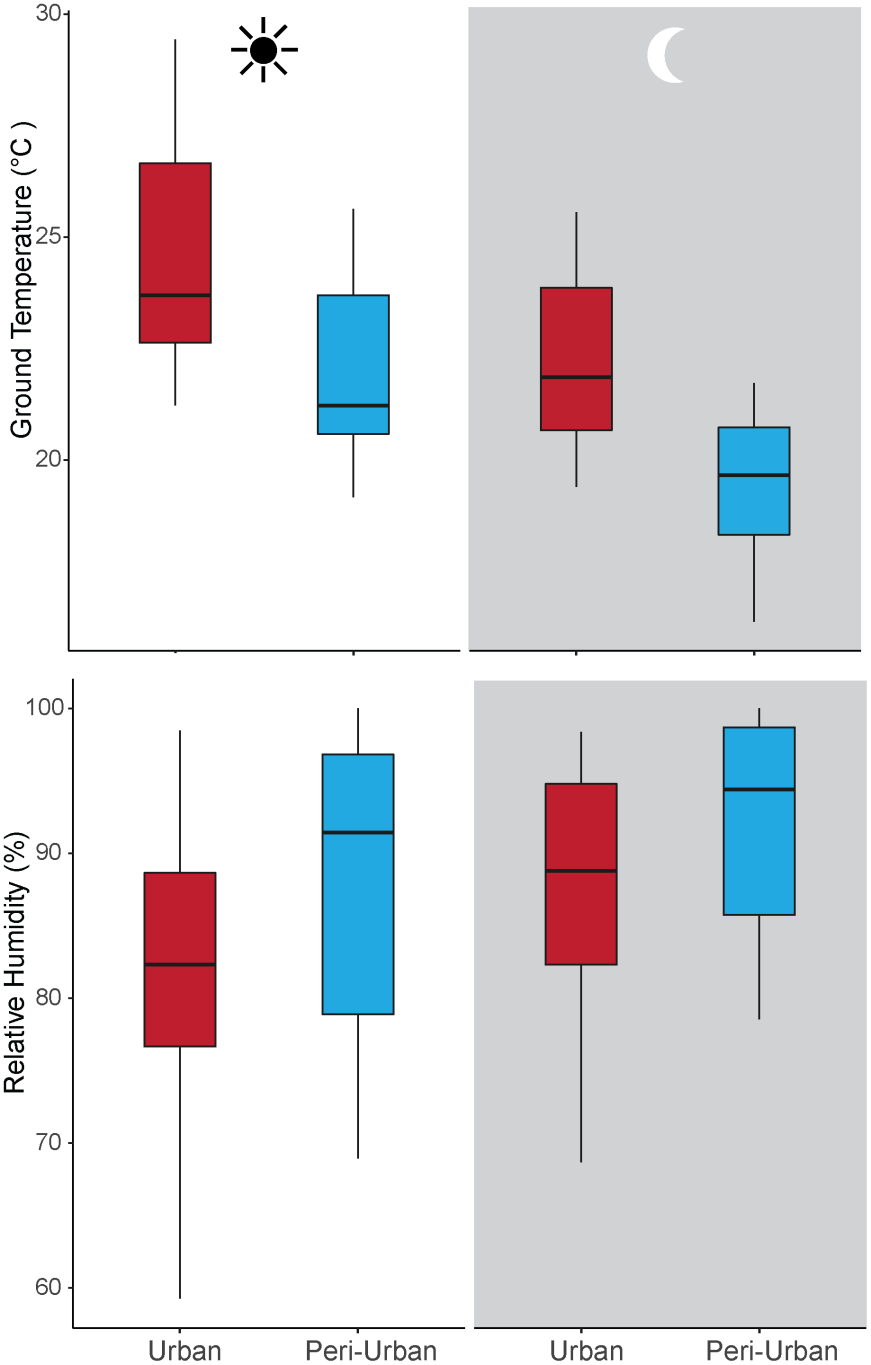
Urban and peri‐urban parks during the day and night differ in ground temperature (top panel) and relative humidity (bottom panel) recorded using data loggers.

Urban sites were on average warmer and drier than peri‐urban sites. Urban parks had on average 2.6°C higher daily temperatures in July compared to the peri‐urban sites (Figure 1, mean ± SE: 24.5 ± 0.4 vs. 21.9 ± 0.3°C) and this difference was maintained during the night (22.3 ± 0.3 vs. 19.4 ± 0.3°C). Both site type (urban vs. peri‐urban: *χ* ^*2*^ = 68.2, *df* = 1, *p* < .001), and time of day (*χ* ^*2*^ = 51.1, *df* = 1, *p* < .001) were significant predictors of the ground temperature measured with data loggers. Relative humidity differences were not as pronounced as temperature differences, but relative humidity (RH) was on average 5.4% higher in peri‐urban habitats (Figure 1, *χ* ^*2*^ = 10, *df* = 1, *p* = .0016) and during the night in both urban and peri‐urban habitats (Figure 1; *χ* ^*2*^ = 10.2, *df* = 1, *p* = .0014).

### 3.2 Thermal tolerance differences

**FIGURE 2:**
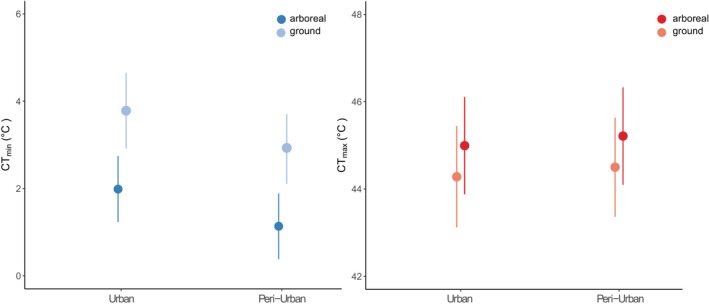
Model predictions of critical thermal limits across urban and peri‐urban sites and habitat types. Critical thermal minimum (CT_min_) is shown in blue and critical thermal maximum (CT_max_) in red. Arboreal ants are always shown in a darker hue.

**FIGURE 3:**
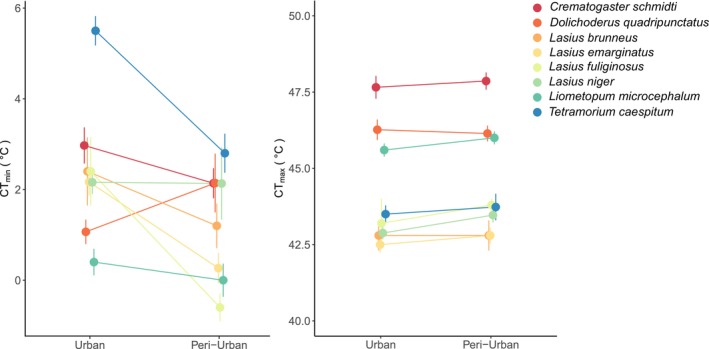
Critical thermal limits across eight species that were found in both urban and peri urban and peri‐urban sites. Critical thermal minimum (CT_min_) was significantly lower in peri‐urban habitats (*χ* ^*2*^ = 11.6, *df* = 1, *p* = .0007); while critical thermal maximum (CT_max_) did not differ across habitat types (*χ* ^*2*^ = 2.8, *df* = 1, *p* = .1).

Critical thermal minimum of ants in urban sites was on average higher compared to ants from peri‐urban sites (Figure 2; *χ* ^*2*^ = 25.7, *df* = 1, *p* = .0009). Arboreal ants had lower CT_min_ compared to ground nesting ants in both urban and peri‐urban sites (*χ* ^*2*^ = 7.6, *df* = 1, *p* = .006). The critical thermal maximum did not vary between urban and peri‐urban sites (Figure 2, *χ* ^*2*^ = 1.3, *df* = 1, *p* = .25), or between arboreal and ground nesting ants (*χ* ^*2*^ = 0.8, *df* = 1, *p* = .366). The set of species for which we measured CTL includes 40% (8/20) species collected in pitfalls in the 3 years of sampling and 75% (6/8) of all species present at baits during the 3 years of sampling (Table S1). We compared community level CT_max_ (Figure 2), but the pattern of no change in CT_max_ and lower CT_min_ in peri‐urban habitats was the same when we examined species level differences (Figure 3).

### 3.3 Abundance and richness changes

Ant abundance in pitfalls varied across sampling years (Figure S2; *χ* ^*2*^ = 10.3, *df* = 2, *p* = .0059) and temperatures (*χ* ^*2*^ = 4.1, *df* = 1, *p* = .0443). Specifically, ant abundance was lower in 2020, compared to two other years although these effects were only marginally significant when years were compared via pairwise comparisons. There is only a trend towards abundance differences between urban and peri‐urban sites (*χ* ^*2*^ = 2.9, *df* = 1, *p* = .089). Ant species richness did not change across sites during 3 years of sampling (*p* = .18) or with temperature (*p* = .86). Instead, wooded area was still the best predictor of species richness (*χ* ^*2*^ = 19.0, *df* = 7, *P* = .008).

### 3.4 Recruitment and occurrence at baits

Urban parks had 9.6 times higher recruitment to baits than peri‐urban sites (*χ* ^*2*^ = 7.3, *df* = 1, *p* = .007), especially ants with higher CT_min_ which were more abundant at baits (*χ* ^*2*^ = 7.3, *df* = 1, *p* = .0068). Sampling year and average surface temperature did not influence ant recruitment at baits (*p*_year_ = .457, *p*_temp_ = .391). *Tetramorium caespitum* was the most abundant ant in urban sites, accounting for 81% of all ants collected on baits. At the same time, *T. caespitum* was completely absent from peri‐urban baits which were dominated by *Lasius niger*, comprising 47% of all collected ants. *Lasius niger* was also present on baits in urban sites, but in low abundance (11% of all ants). Ant occurrence was best predicted by the sampling year (*χ* ^*2*^ = 10.1, *df* = 2, *p* = .0063) as it was higher in 2021 compared to 2020, the only significant difference observed among years after pairwise comparisons (Figure S3). Neither average surface temperature (*χ* ^*2*^ = 2.2, *df* = 1, *p* = .135) nor site were good predictors of ant occurrence at baits (*χ* ^*2*^ = 0.11, *df* = 1, *p* = .731).

## 4 DISCUSSION

Our study is the first to test community level differences in the thermal tolerance of urban ants, and we found that cold, and not heat tolerance depends on habitat temperatures. Urban sites were warmer and drier compared to peri‐urban sites and yet, despite 2.6°C higher average temperature in urban sites, heat tolerances of ants in them were comparable to the ones in peri‐urban sites. Instead, cold tolerance varied predictably with habitat temperature, as ants in cooler, peri‐urban sites, tolerated lower temperatures. Our finding suggests that in temperate zones, cold pressure might still be stronger than heat pressure in urban habitats, as was recently found for five North American ants (Youngsteadt et al., 2023). This finding is further supported by the fact that ant recruitment across 3 years of sampling was best predicted by cold, and not heat tolerance. Additionally, we discovered lower ant abundance in a year with a lower average temperature, suggesting that temperature still governs ant foraging.

### 4.1 Thermal tolerance across urban habitats

Invariant CT_max_ that we recorded across the urban landscape does not support the thermal adaptation hypothesis. It also disagrees with previous findings from the tropical urban–rural comparison, which found higher heat tolerance in urban leaf cutting ant populations (Angilletta et al., 2007). Temperate zone ants like seed‐harvesting ants (Warren et al., 2018), acorn ants (Diamond et al., 2017) and North American woodland ants (Diamond & Martin, 2021) have higher CT_max_ in urban habitats, and CT_min_ of these ants also follows habitat temperature, being higher in warmer, more urbanized sites. In contrast, chill coma recovery time between urban and rural populations did not differ (Angilletta et al., 2007), likely because tropical ants do not experience significant cold pressure in their habitat. Therefore, selection pressures on thermal tolerance of ectotherms between tropical and temperature urban habitats likely differ.

There are two main reasons why our CT_max_ results could differ from some of the previous studies. First, Zagreb might have a weaker urban heat island effect than metropolis cities like Sao Paulo, where observed heat tolerance was higher in urban than rural sites (Angilletta et al., 2007). Overall, the climates across these two cities differ substantially. The daily mean temperature in Sao Paulo is 19.6°C (Roca‐Barceló et al., 2022), while the mean annual temperature in Zagreb is 12.1°C (Bonacci et al., 2021). Winters in Sao Paulo City (from June to August) range in monthly mean temperature from 14.4°C to 16.4°C, while the mean monthly winter temperature in Zagreb is 2.5°C (from December to February) (Bonacci et al., 2021). Thus, the overall climate is warmer in Sao Paulo, particularly during the winter times, which are much cooler in Zagreb.

Second, most of our ants were arboreal, so they might already have adapted to warmer habitats. Other studies on Hymenoptera found the same pattern as our study, for example, CT_min_ of honeybees was higher in urban areas (lower cold tolerance than peri‐urban honeybees) and as a result, narrower T_ranges_ were found in the city as their CT_max_ did not vary with urbanization (Sánchez‐Echeverría et al., 2019). Higher variability in CT_min_ than CT_max_, and higher CT_min_ (lower cold tolerance) in urban compared to rural areas were also found in one species of acorn ants (Diamond et al., 2017). Together, this suggests that different evolutionary forces might be acting on organisms in the urbanized landscape than in semi‐natural or rural areas. Our results on invariant CT_max_ confirms a recent finding across urban–rural gradient in five temperate ant species (Youngsteadt et al., 2023) indicating that temperate zone ants did not adapt nor acclimatize CT_max_ to the urban heat island effect, as did the tropical ants.

We recorded higher average daily temperatures in urban habitats during the three‐year period of this study, and even temperature maxima and minima are higher in urban environments. But the long‐term temperature records (58‐years of measurements) show that urban and peri‐urban habitats in Zagreb do not differ with respect to the maximum temperature, while the temperature minima are significantly lower in peri‐urban habitats (Nimac et al., 2021). This suggests that ants' thermal limits across this urban environment are adjusted to the differences in thermal minima experienced in the habitat. Thus, thermal tolerances might be governed by environmental thermal extremes, not environmental temperature averages, a pattern previously recorded at large scales (Bujan et al., 2020; Diamond & Chick, 2018).

At the habitat level, we found the same pattern, of invariant CT_max_ and lower CT_min_ in the canopy compared to the ground nesting ants. CT_max_ was remarkably consistent at both the community level (Figure 2) and the species level (Figure 3). In contrast, lower CT_min_ was found in peri‐urban sites at the community level, and species level, as most of the species that we recorded across urban and peri‐urban sites had lower CT_min_ in peri‐urban habitats. This suggests that intraspecific variation in CT_min_, and not species identity, is most likely driving the difference in cold tolerance across the sites.

The majority of the shared species that occurred in both urban and peri‐urban sites (Figure 3) are arboreal (6/8) and arboreal ants are generally well adapted to hot and dry environments (Bujan et al., 2016), so cold exposure could be more detrimental for the ants in the temperate canopy. Our study, as studies on tropical ant communities, found wider thermal ranges in arboreal compared to ground nesting ants (Kaspari et al., 2015; Leahy et al., 2021). However, in both of those tropical studies, CT_max_ was higher in the canopy than the understory, which was not the case in our study. Canopy in urban habitat does not seem to impose additional heat stress compared to the understory of urban parks. Instead, they might offer a cooler microclimate in the city, more nesting sites, and habitat with less disturbances (e.g., mowing, planting, pesticide treatment) that could result in denser aphid populations providing honeydew for aphid‐tending ants. Thus, canopy habitats might be increasingly important for maintaining and preserving biodiversity of urban ants.

### 4.2 Abundance and richness changes in urban ant communities

The differences in temperature among sites, based on weather station data, did not impact overall ant richness, consistent with previous studies (Bujan et al., 2022; Pelini et al., 2011), suggesting that this community feature is quite resistant to thermal stress. Previously, we found that richness in these Zagreb parks is predominantly driven by the amount of wooded area (Ješovnik & Bujan, 2021), so we would expect to observe the same pattern using microclimates.

Ant abundance was lower in 2020, likely because the average monthly temperature in 2020 (20.7°C) was significantly lower than in both 2019 (24.5°C) and 2021 (23.9°C). Thus, ant activity is strongly impacted by the thermal environment in urban, as well as natural habitats (Cerdá et al., 1998). Higher temperatures observed in urban habitats might promote higher foraging activity which could have beneficial effects for colonies of urban ants acquiring more resources.

### 4.3 Activity changes in urban ant communities

Urban ants had higher rate of recruitment, compared to the peri‐urban ants, even though urban community was less species rich, and more homogeneous, consistent with previous findings (Savage et al., 2015). Urban habitats are often dominated by the pavement ant (*T. caespitum*) which heavily relies on human generated waste (Penick et al., 2015; Savage et al., 2015), and we found dominance of pavement ants in our urban parks. This species is likely driving our modeled estimate of CT_min_ as a good predictor of ant recruitment, because of its low cold tolerance (high CT_min_, Figure 3) and high recruitment on baits in urban parks.

Baiting is an active collecting method that targets common and dominant ants which are generalists and thus imitates natural foraging to resources (Bestelmeyer et al., 2000). Often the bait will be occupied by the species that recruits fast, in large numbers, and aggressively defends the bait (Bujan et al., 2016). Pitfall traps are not selective, they passively collect ants over longer time periods, which is why pitfalls recorded a higher number of species than baits. Both methods are standard methods for ant sampling (Agosti et al., 2000) and we combined them for a comprehensive understanding of community level changes and activity changes across sites. Although baits and pitfalls attracted different number of species, in both cases, abundance was lower in 2020. Moreover, there was no change in species richness over the years, suggesting that, regardless of the different approach, they record the same community level changes.

Ant occurrence at baits was the highest in 2021—somewhat similar to abundance (activity density) in pitfalls which was higher in 2021 and 2022, compared to 2020. And while we could explain differences in abundance in pitfalls with temperature, surprisingly, the temperature measured at the time of bating did not predict ant occurrence (species presence or absence at baits). Preferred foraging temperatures of ants did not change along urban thermal gradients (Youngsteadt et al., 2023), which suggests that species specific activity patterns might not be flexible enough to change with warming in the cities, or the warming is still not stressful enough. Taken together, our results suggest that we need to rethink predictions about urban heat islands driving insect adaptations, as not all insects will increase heat tolerance in urban habitats, and cold tolerance might be a more variable or adaptable trait, particularly in the temperate zone.

## AUTHOR CONTRIBUTIONS

**Jelena Bujan:** Conceptualization (equal); data curation (equal); formal analysis (lead); funding acquisition (supporting); investigation (equal); methodology (equal); project administration (supporting); writing – original draft (lead); writing – review and editing (lead). **Cleo Bertelsmeier:** Conceptualization (supporting); formal analysis (supporting); funding acquisition (lead); writing – original draft (supporting); writing – review and editing (supporting). **Ana Ješovnik:** Conceptualization (equal); data curation (equal); formal analysis (supporting); funding acquisition (lead); investigation (equal); methodology (equal); project administration (lead); writing – original draft (equal); writing – review and editing (equal).

## Supporting information


Appendix S1

## Data Availability

Data supporting the findings of this study are provided for peer review and will be publicly available in Figshare upon publication https://figshare.com/s/b466915a1d1ce115d441.

## References

[ece311335-bib-0001] Agosti, D. , Majer, J. D. , Alonso, L. E. , & Schultz, T. R. (2000). Standard methods for measuring and monitoring biodiversity. Smithsonian institution press.

[ece311335-bib-0002] Alberti, M. , Correa, C. , Marzluff, J. M. , Hendry, A. P. , Palkovacs, E. P. , Gotanda, K. M. , Hunt, V. M. , Apgar, T. M. , & Zhou, Y. (2017). Global urban signatures of phenotypic change in animal and plant populations. Proceedings of the National Academy of Sciences of the United States of America, 114, 8951–8956. 28049817 10.1073/pnas.1606034114 PMC5576774

[ece311335-bib-0003] Angilletta, M. J. J. , Wilson, R. S. , Niehaus, A. C. , Sears, M. W. , Navas, C. A. , & Ribeiro, P. L. (2007). Urban physiology: City ants possess high heat tolerance. PLoS One, 2, e258. 17327918 10.1371/journal.pone.0000258 PMC1797824

[ece311335-bib-0004] Aram, F. , Higueras García, E. , Solgi, E. , & Mansournia, S. (2019). Urban green space cooling effect in cities. Heliyon, 5, e01339. 31008380 10.1016/j.heliyon.2019.e01339 PMC6458494

[ece311335-bib-0005] Bestelmeyer, B. T. , Agosti, D. , Alonso, L. E. , Brandao, C. R. F. , Brown, W. L., Jr. , Delabie, J. H. C. , & Silvestre, R. (2000). Field Techniques for the Study of Ground‐Dwelling Ants. In Ants: Staandard methods for measuring and monitoring biodiversity (pp. 122–144). Smithsonian Institution Press.

[ece311335-bib-0006] Bonacci, O. , Bonacci, D. , & Roje‐Bonacci, T. (2021). Different air temperature changes in continental and Mediterranean regions: a case study from two Croatian stations. Theoretical and Applied Climatology, 145, 1333–1346.

[ece311335-bib-0007] Boyle, M. J. W. , Bishop, T. R. , Luke, S. H. , van Breugel, M. , Evans, T. A. , Pfeifer, M. , Fayle, T. M. , Hardwick, S. R. , Lane‐Shaw, R. I. , Yusah, K. M. , Ashford, I. C. R. , Ashford, O. S. , Garnett, E. , Turner, E. C. , Wilkinson, C. L. , Chung, A. Y. C. , & Ewers, R. M. (2021). Localised climate change defines ant communities in human‐modified tropical landscapes. Functional Ecology, 35, 1094–1108.

[ece311335-bib-0008] Brooks, M. E. , Kristensen, K. , van Benthem, K. J. , Magnusson, A. , Berg, C. W. , Nielsen, A. , Skaug, H. J. , Mächler, M. , & Bolker, B. M. (2017). glmmTMB balances speed and flexibility among packages for zero‐inflated generalized linear mixed modeling. R Journal, 9, 378–400.

[ece311335-bib-0009] Bujan, J. , Nottingham, A. T. , Velasquez, E. , Meir, P. , Kaspari, M. , & Yanoviak, S. P. (2022). Tropical ant community responses to experimental soil warming. Biology Letters, 18, 3–7. 10.1098/rsbl.2021.0518 PMC8984296 35382584

[ece311335-bib-0010] Bujan, J. , Roeder, K. A. , Beurs, K. , Weiser, M. D. , & Kaspari, M. (2020). Thermal diversity of north American ant communities: Cold tolerance but not heat tolerance tracks ecosystem temperature. Global Ecology and Biogeography, 29, 1486–1494.

[ece311335-bib-0011] Bujan, J. , Yanoviak, S. P. , & Kaspari, M. (2016). Desiccation resistance in tropical insects: Causes and mechanisms underlying variability in a Panama ant community. Ecology and Evolution, 6, 6282–6291. 27648242 10.1002/ece3.2355 PMC5016648

[ece311335-bib-0012] Cerdá, X. , Retana, J. , & Cros, S. (1998). Critical thermal limits in Mediterranean ant species: Trade‐off between mortality risk and foraging performance. Functional Ecology, 12, 45–55.

[ece311335-bib-0013] Deilami, K. , Kamruzzaman, M. , & Liu, Y. (2018). Urban heat Island effect: A systematic review of spatio‐temporal factors, data, methods, and mitigation measures. International Journal of Applied Earth Observation and Geoinformation, 67, 30–42.

[ece311335-bib-0014] Diamond, S. , Nichols, L. , McCoy, N. , Hirsch, C. , Pelini, S. , Sanders, N. J. , Ellison, A. M. , Gotelli, N. J. , & Dunn, R. R. (2012). A physiological trait‐based approach to predicting the responses of species to experimental climate warming. Ecology, 93, 2305–2312. 23236901 10.1890/11-2296.1

[ece311335-bib-0015] Diamond, S. E. , Chick, L. , Perez, A. , Strickler, S. A. , & Martin, R. A. (2017). Rapid evolution of ant thermal tolerance within an urban heat Island. Biological Journal of the Linnean Society, 121, 248–257.

[ece311335-bib-0016] Diamond, S. E. , & Chick, L. D. (2018). The Janus of macrophysiology: Stronger effects of evolutionary history, but weaker effects of climate on upper thermal limits are reversed for lower thermal limits in ants. Current Zoology, 64, 223–230. 30402063 10.1093/cz/zox072 PMC5905527

[ece311335-bib-0017] Diamond, S. E. , & Martin, R. A. (2021). Physiological adaptation to cities as a proxy to forecast global‐scale responses to climate change. Journal of Experimental Biology, 224, jeb229336. 33627462 10.1242/jeb.229336

[ece311335-bib-0018] Fattorini, S. , & Galassi, D. M. P. (2016). Role of urban green spaces for saproxylic beetle conservation: a case study of tenebrionids in Rome, Italy. Journal of Insect Conservation, 20, 737–745.

[ece311335-bib-0019] Hall, D. M. , Camilo, G. R. , Tonietto, R. K. , Ollerton, J. , Ahrné, K. , Arduser, M. , Ascher, J. S. , Baldock, K. C. R. , Fowler, R. , Frankie, G. , Goulson, D. , Gunnarsson, B. , Hanley, M. E. , Jackson, J. I. , Langellotto, G. , Lowenstein, D. , Minor, E. S. , Philpott, S. M. , Potts, S. G. , … Threlfall, C. G. (2017). The city as a refuge for insect pollinators. Conservation Biology, 31, 24–29. 27624925 10.1111/cobi.12840

[ece311335-bib-0020] Harting, F. (2021). *DHARMa: Residual diagnostics for hierarchical (multi‐level / mixed) regression models*. R Package Version 0.4.4. https://CRAN.R‐project.org/package=DHARMa. https://cran.r‐project.org/package=DHARMa

[ece311335-bib-0021] Ješovnik, A. , & Bujan, J. (2021). Wooded areas promote species richness in urban parks. Urban Ecosystems, 24, 1305–1315.

[ece311335-bib-0022] Kaspari, M. , Clay, N. A. , Lucas, J. , Yanoviak, S. P. , & Kay, A. (2015). Thermal adaptation generates a diversity of thermal limits in a rainforest ant community. Global Change Biology, 21, 1092–1102. 25242246 10.1111/gcb.12750

[ece311335-bib-0023] Leahy, L. , Scheffers, B. R. , Andersen, A. N. , Hirsch, B. T. , & Williams, S. E. (2021). Vertical niche and elevation range size in tropical ants: Implications for climate resilience. Diversity and Distributions, 27, 485–496.

[ece311335-bib-0024] Lenth, R. , Singmann, H. , Love, J. , Buerkner, P. , & Herve, M. (2020). Package ‘emmeans’ R topics documented : R Package Version 1.15–15.

[ece311335-bib-0025] Lutterschmidt, W. I. , & Hutchison, V. H. (1997). The critical thermal maximum: History and critique. Canadian Journal of Zoology, 75, 1561–1574.

[ece311335-bib-0026] Martin, R. A. , Chick, L. D. , Yilmaz, A. R. , & Diamond, S. E. (2019). Evolution, not transgenerational plasticity, explains the adaptive divergence of acorn ant thermal tolerance across an urban—rural temperature cline. Evolutionary Applications, 12, 1678–1687. 31462922 10.1111/eva.12826 PMC6708418

[ece311335-bib-0027] Merckx, T. , Kaiser, A. , & Van Dyck, H. (2018). Increased body size along urbanization gradients at both community and intraspecific level in macro‐moths. Global Change Biology, 24, 3837–3848. 29791767 10.1111/gcb.14151

[ece311335-bib-0028] Morecroft, M. D. , Taylor, M. E. , & Oliver, H. R. (1998). Air and soil microclimates of deciduous woodland compared to an open site. Agricultural and Forest Meteorology, 90, 141–156.

[ece311335-bib-0029] Nimac, I. , Herceg‐Bulić, I. , Cindrić Kalin, K. , & Perčec Tadić, M. (2021). Changes in extreme air temperatures in the mid‐sized European city situated on southern base of a mountain (Zagreb, Croatia). Theoretical and Applied Climatology, 146, 429–441.

[ece311335-bib-0030] Pelini, S. L. , Boudreau, M. , McCoy, N. , Ellison, A. M. , Gotelli, N. J. , Sanders, N. J. , & Dunn, R. R. (2011). Effects of short‐term warming on low and high latitude forest ant communities. Ecosphere, 2, 1–12.

[ece311335-bib-0031] Penick, C. A. , Savage, A. M. , & Dunn, R. R. (2015). Stable isotopes reveal links between human food inputs and urban ant diets. Proceedings of the Royal Society B: Biological Sciences, 282, 20142608. 10.1098/rspb.2014.2608 PMC4426608 25833850

[ece311335-bib-0032] Pinho, P. , Correia, O. , Lecoq, M. , Munzi, S. , Vasconcelos, S. , Gonçalves, P. , Rebelo, R. , Antunes, C. , Silva, P. , Freitas, C. , Lopes, N. , Santos‐Reis, M. , & Branquinho, C. (2016). Evaluating green infrastructure in urban environments using a multi‐taxa and functional diversity approach. Environmental Research, 147, 601–610. 26777032 10.1016/j.envres.2015.12.025

[ece311335-bib-0033] R Core Team . (2023). R: A language and environment for statistical computing. R Foundation for statistical computing.

[ece311335-bib-0034] Radchenko, A. G. , & Elmes, G. W. (2010). Myrmica ants (Hymenoptera: Formicidae) of the Old World. Page Fauna Mundi.

[ece311335-bib-0035] Roca‐Barceló, A. , Fecht, D. , Pirani, M. , Piel, F. B. , Nardocci, A. C. , & Vineis, P. (2022). Trends in temperature‐associated mortality in São Paulo (Brazil) between 2000 and 2018: An example of disparities in adaptation to cold and heat. Journal of Urban Health, 99, 1012–1026. 36357626 10.1007/s11524-022-00695-7 PMC9727050

[ece311335-bib-0036] Roeder, K. A. , Roeder, D. V. , & Bujan, J. (2021). Ant thermal tolerance: A review of methods, hypotheses, and sources of variation. Annals of the Entomological Society of America, 114, 459–469.

[ece311335-bib-0037] Roeder, K. A. , Roeder, D. V. , & Kaspari, M. (2018). The role of temperature in competition and persistence of an invaded ant assemblage. Ecological Entomology, 43, 774–781.

[ece311335-bib-0038] Sánchez‐Echeverría, K. , Castellanos, I. , Mendoza‐Cuenca, L. , Zuria, I. , & Sánchez‐Rojas, G. (2019). Reduced thermal variability in cities and its impact on honey bee thermal tolerance. PeerJ, 2019, 1–17. 10.7717/peerj.7060 PMC6557256 31211017

[ece311335-bib-0039] Savage, A. M. , Hackett, B. , Guénard, B. , Youngsteadt, E. K. , & Dunn, R. R. (2015). Fine‐scale heterogeneity across Manhattan's urban habitat mosaic is associated with variation in ant composition and richness. Insect Conservation and Diversity, 8, 216–228.

[ece311335-bib-0040] Seifert, B. (2018). The ants of central and North Europe. Lutra Verlags Und Vertriebsgesellschaft.

[ece311335-bib-0041] Seto, K. C. , Güneralp, B. , & Hutyra, L. R. (2012). Global forecasts of urban expansion to 2030 and direct impacts on biodiversity and carbon pools. Proceedings of the National Academy of Sciences of the United States of America, 109(16083–16), 88. 10.1073/pnas.1211658109 PMC3479537 22988086

[ece311335-bib-0042] Šiško, D. , & Polančec, V. (2019). Statistički ljetopis grada Zagreba. Zagreb .

[ece311335-bib-0043] Sunday, J. M. , Bates, A. E. , Kearney, M. R. , Colwell, R. K. , Dulvy, N. K. , Longino, J. T. , & Huey, R. B. (2014). Thermal‐safety margins and the necessity of thermoregulatory behavior across latitude and elevation. Proceedings of the National Academy of Sciences of the United States of America, 111, 5610–5615. 24616528 10.1073/pnas.1316145111 PMC3992687

[ece311335-bib-0044] Szulkin, M. , Munshi‐South, J. , & Charmantier, A. (2020). Urban evolutionary biology. Oxford University Press.

[ece311335-bib-0045] Theodorou, P. , Baltz, L. M. , Paxton, R. J. , & Soro, A. (2021). Urbanization is associated with shifts in bumblebee body size, with cascading effects on pollination. Evolutionary Applications, 14, 53–68. 33519956 10.1111/eva.13087 PMC7819558

[ece311335-bib-0046] Wagner, H. C. , Arthofer, W. , Seifert, B. , Muster, C. , Steiner, F. M. , & Schlick‐Steiner, B. (2017). Light at the end of the tunnel: Integrative taxonomy delimits cryptic species in the *Tetramorium caespitum* complex (Hymenoptera: Formicidae). Myrmecological News, 25, 95–129.

[ece311335-bib-0047] Warren, R. J. , Bayba, S. , & Krupp, K. T. (2018). Interacting effects of urbanization and coastal gradients on ant thermal responses. Journal of Urban Ecology, 4, 1–11.

[ece311335-bib-0048] Youngsteadt, E. , Henderson, R. C. , Savage, A. M. , Ernst, A. F. , Dunn, R. R. , & Frank, S. D. (2015). Habitat and species identity, not diversity, predict the extent of refuse consumption by urban arthropods. Global Change Biology, 21, 1103–1115. 25463151 10.1111/gcb.12791

[ece311335-bib-0049] Youngsteadt, E. , Prado, S. G. , Keleher, K. J. , & Kirchner, M. (2023). Can behaviour and physiology mitigate effects of warming on ectotherms? A test in urban ants. Journal of Animal Ecology, 92, 568–579. 36642830 10.1111/1365-2656.13860

[ece311335-bib-0050] Zaninovic, K. , Gajić‐Capka, M. , & Perčec Tadić, M. (2008). Klimatski atlas Hrvatske/Climate atlas of Croatia 1961–1990., 1971–2000. Zagreb .

