# Supplementary material for: Insects in temperate urban parks face stronger selection pressure from the cold than the heat
Source: Ecol Evol. 2024 Aug 19;14(8):e11335. doi: 10.1002/ece3.11335 (PMC11333530; doi:10.1002/ece3.11335)
Supplement: Supplementary file 1 — Appendix S1 [file ECE3-14-e11335-s001.docx]

**Supplementary Information to:**

Bujan J, Bertelsmeier C, Ješovnik A. Insects in temperate urban parks face stronger selection pressure from the cold than the heat

Appendinx S1 Contents:

Figure S1. Map of all sampled sites

Figure S2 Ant abundance in pitfalls across three years of sampling.

Figure S3 Occurrence of ants at baits across three years of sampling.

Table S1. List of collected and tested species

*
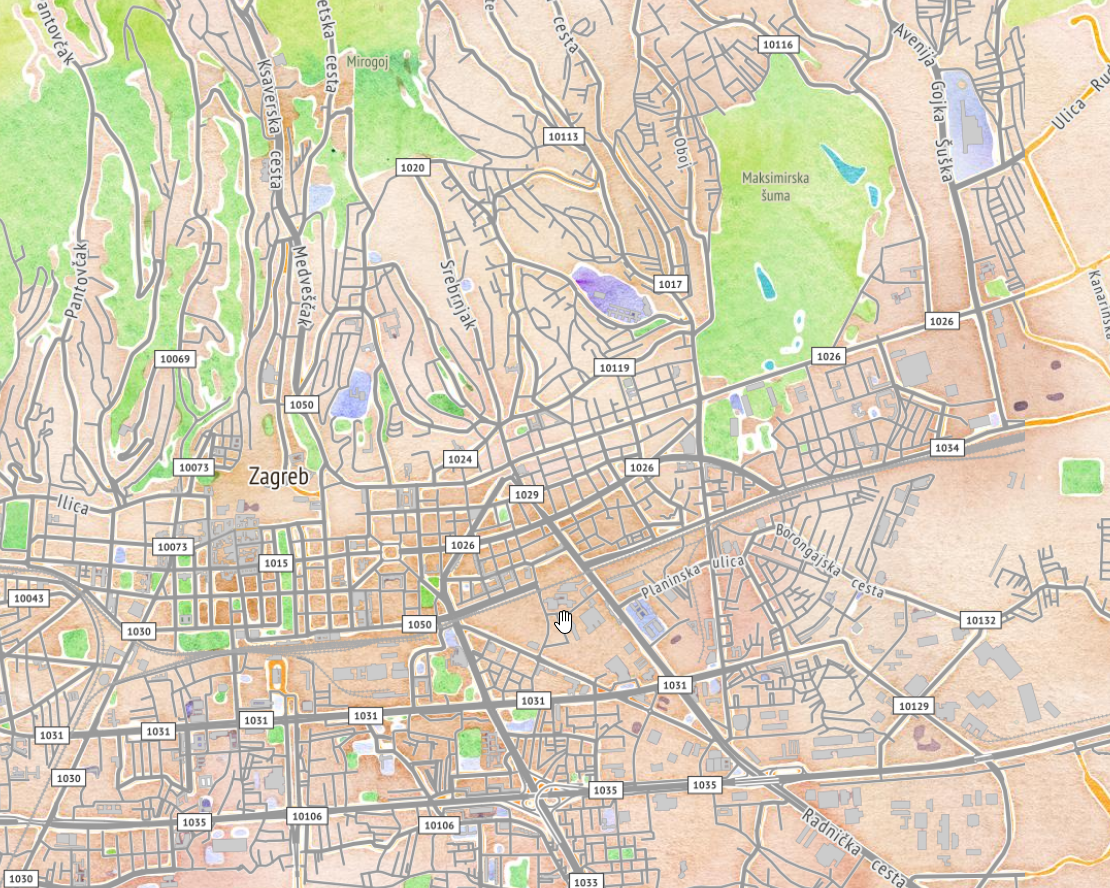
*­­­­

Figure S1. Sites at which we measured: temperatures (white dots), critical thermal limits (rectangles - red in urban, blue peri-urban parks), and ant activity (all dots). Three localities were within a large protected forested area at the edge of the city (peri-urban sites) and five were in the city center and we refer to those as urban sites.


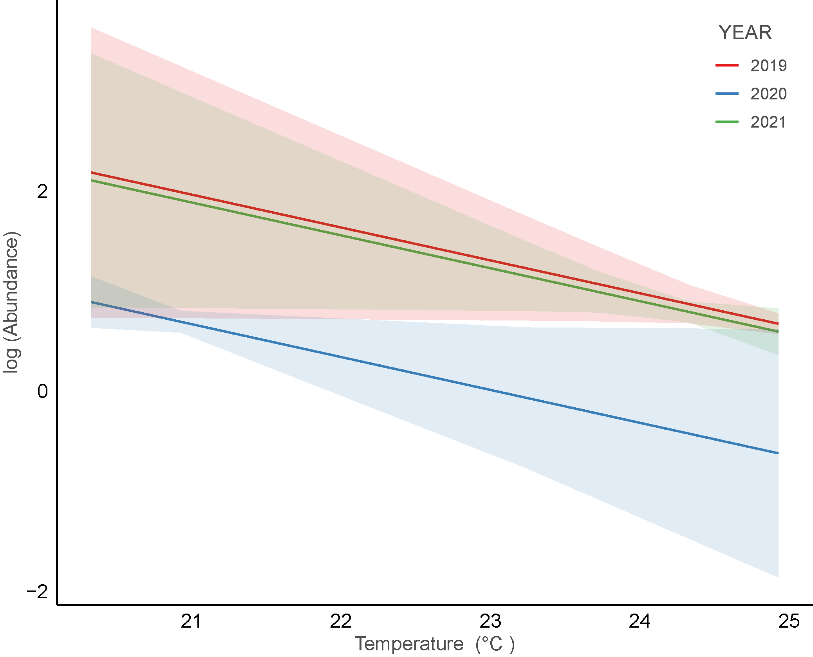


Figure S2 Differences in ant abundance in pitfalls across three years of sampling.


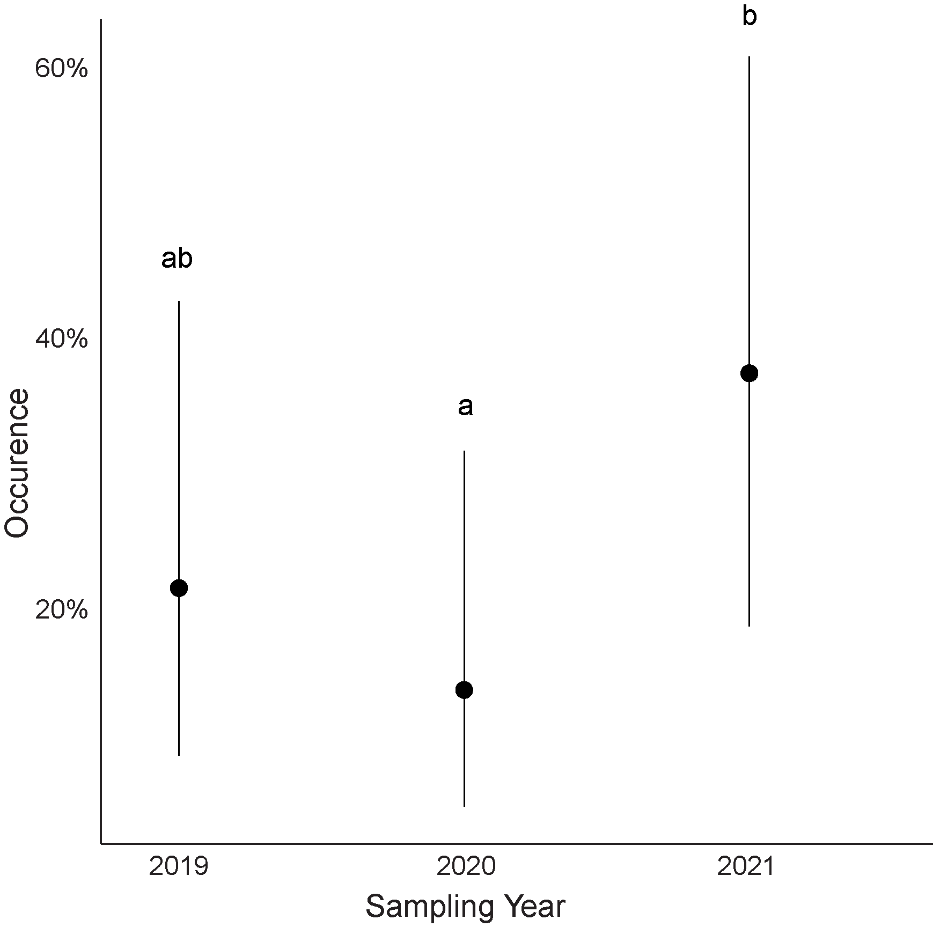


Figure S3 Occurrence of ants at baits across three years of sampling.

Table S1. Complete set of species collected in pitfalls and baits during three years of sampling. Critical thermal limits (CTL) column indicates presence (1) or absence of ramping trails conducted for 14 species.

|  |  |  |  |
| --- | --- | --- | --- |
| SPECIES | CTL | BAIT | PITFALL |
| *Aphaenogaster subterranea* | 0 | 0 | 1 |
| *Camponotus fallax* | 1 | 0 | 0 |
| *Crematogaster schmidti* | 1 | 1 | 1 |
| *Colobopsis truncata* | 1 | 0 | 0 |
| *Dolichoderus quadripunctatus* | 1 | 0 | 0 |
| *Formica cunicularia* | 1 | 1 | 1 |
| *Formica fusca* | 0 | 0 | 1 |
| *Formica gagates* | 0 | 0 | 1 |
| *Formica lemani* | 1 | 0 | 0 |
| *Formica rufibarbis* | 0 | 0 | 1 |
| *Lasius brunneus* | 1 | 0 | 0 |
| *Lasius emarginatus* | 1 | 0 | 1 |
| *Lasius flavus* | 0 | 0 | 1 |
| *Lasius fuliginosus* | 1 | 0 | 0 |
| *Lasius myops* | 0 | 0 | 1 |
| *Lasius niger* | 1 | 1 | 1 |
| *Liometopum microcephalum* | 1 | 0 | 1 |
| *Myrmecina graminicola* | 0 | 0 | 1 |
| *Myrmica curvithorax* | 0 | 1 | 1 |
| *Myrmica scabrinodis* | 0 | 1 | 1 |
| *Plagiolepis pygmeae* | 1 | 1 | 1 |
| *Prenolepis nitens* | 0 | 0 | 1 |
| *Solenopsis fugax* | 0 | 0 | 1 |
| *Tapinoma subboreale* | 0 | 0 | 1 |
| *Temnothorax crassispinus* | 1 | 1 | 1 |
| *Tetramorium caespitum* | 1 | 1 | 1 |
| **Total species number** | **14** | **8** | **20** |
